# Early feeding practices and consumption of ultraprocessed foods at 6 y of age: Findings from the 2004 Pelotas (Brazil) Birth Cohort Study

**DOI:** 10.1016/j.nut.2017.09.012

**Published:** 2018-03

**Authors:** Renata M. Bielemann, Leonardo Pozza Santos, Caroline dos Santos Costa, Alicia Matijasevich, Iná S. Santos

**Affiliations:** a Post-Graduate Program in Epidemiology, Federal University of Pelotas, Brazil; b Nutrition Department, Federal University of Pelotas, Brazil; c Nutrition School, Federal University of Pampa, Brazil; d Department of Preventive Medicine, School of Medicine, University of São Paulo, Brazil

**Keywords:** Ultraprocessed foods, Complementary feeding, Breastfeeding, Cohort studies

## Abstract

**Objective:**

The aim of this study was to examine the association between early feeding practices and consumption of ultraprocessed foods in children at age 6 y.

**Methods:**

This was a prospective cohort study using data from 3427 children who participated in the 2004 Pelotas Cohort Study and who had available food frequency questionnaire information at 6 y. Information about exclusive and total breastfeeding duration as well as age at introduction of semisolid and solid foods was used to define early feeding practices. Consumption of ultraprocessed foods was defined as proportion of total daily energy intake that came from ultraprocessed foods at age 6 y. Crude and adjusted linear regression models were employed to analyze the effect of early feeding practices on ultraprocessed foods consumption.

**Results:**

It was determined that 40.3% of total daily energy intake at 6 y came from ultraprocessed foods. In crude linear regression models, early feeding practices (exclusive and total breastfeeding duration, and age at introduction of semisolid and solid foods) were negatively associated with ultraprocessed foods consumption. After adjustment, only exclusive breastfeeding duration and age at introduction of solid foods remained associated with consumption of ultraprocessed foods, although the observed effects size was small. Children exclusively breastfed for ≥3 mo and those who had solid foods introduced at ≥4 mo consumed a lower proportion of daily energy intake from ultraprocessed foods.

**Conclusion:**

This study supports the need to promote healthy early feeding practices including the support of breastfeeding to promote healthier eating habits later in childhood.

## Highlights

• Newborn infants from southern Brazil were followed up in infancy and childhood.
• Breastfeeding duration and timing of introduction of foods were recorded.
• Ultraprocessed food intake was evaluated by a food frequency questionnaire.
• Introduction of solid foods was related with consumption of ultraprocessed foods.
• Longer exclusively breastfed infants had lower ultraprocessed food consumption later.

## Introduction

Non-communicable diseases (NCDs) were responsible for 65% (34.5 million) of all worldwide deaths in 2010, with one-fourth occurring in individuals <60 y of age [1]. Smoking, alcohol consumption, physical inactivity, and unhealthy diet are considered important determinants of NCDs [2], [3]. Much has been done in an attempt to prevent the occurrence of such behaviors. Specifically with regard to dietary intake, the last decade has been marked by promotion of policies targeting regulation, taxation, pricing, banning, and restricting advertising and sponsorship of major food industries in several countries due to the role of dietary intake in an increase in NCDs [2], [3].

Strategies employed by food industries to promote sales of ultraprocessed foods (UPFs) have raised concern in the public health sector because of their potential to increase UPF consumption [4]. UPFs are industrially manufactured products that are produced through several processing steps and techniques and labeled as ready to eat, drink, or heat. Due to their high degree of processing, they are extremely palatable and durable. UPF also are characterized by high energy density due to the ingredients used in their production [5], [6], [7], [8].

The UPF household availability has markedly increased over the last decades [4], and their consumption has been associated with higher prevalence of obesity in adolescents and adults [9], [10], [11]. In 2010, the first proposal of foodstuffs classification based on their processing degree was published by Monteiro et al. [5], and was updated in 2016 [6]. The NOVA system is a food classification that takes the nature, extent, and purpose of food processing into account to group foods according to their degree of processing [5], [6], [7], [8], and has been used in studies from several countries.

Despite recent studies associating UPF consumption with obesity, there are few studies investigating the role of early feeding habits on UPF consumption. Early feeding practices have been associated with eating habits during school age [12], [13]. Recent investigations have shown that longer duration of exclusive breastfeeding was positively associated with higher intake of fruits and vegetables [12], [13]. One possible reason for the relationship between breastfeeding and later food consumption is the role of early life sensory experiences on food preferences in infancy and childhood [14]. Additionally, children usually prefer sweet and salty foods (characteristics of UPFs) compared with bitter ones [15], [16].

However, to our knowledge, there is no consensus on whether early introduction of complementary feeding promotes good [17] or harmful [18] nutritional outcomes. Also, there is no evidence regarding the effects of early feeding practices on UPF consumption preference later in childhood. Therefore, we aimed to study the association between early feeding practices and consumption of UPF in children 6 y of age from the 2004 Pelotas (Brazil) Birth Cohort Study.

## Methods

### Participants

Pelotas is a city of 330 000 inhabitants located in southern Brazil. From January 1 to December 31, 2004, a population-based birth cohort study was initiated in Pelotas. In all, 4231 live neonates were recruited to take part in the 2004 Pelotas Birth Cohort Study (99.2% of all births to mothers from urban area of Pelotas). Soon after delivery, trained fieldworkers interviewed mothers. The mothers’ newborn infants were examined within 24 h of delivery (the perinatal study). Cohort members were followed up when they were 3 mo and 1, 2, 4, 6, and 11 y of age, with retention rates of 95.7%, 94.3%, 93.5%, 92%, 90.2%, and 86.6%, respectively. Anthropometric variables as well as information on health status, child development, housing conditions, and socioeconomic characteristics were collected at each follow-up.

A detailed description of methods is given elsewhere [19], [20]. In all follow-ups, mothers or legal guardians gave written consent to participate in the study and the Research Ethics Committee of Medical School of the Federal University of Pelotas approved all follow-up waves.

### Early feeding practices

Information on early feeding practices (exclusive and total breastfeeding duration, and age at introduction of semisolid and solid foods) was gathered in 3- and 12-mo follow-ups. According to the World Health Organization [21], breastfeeding was considered exclusive when children's feeding practices were only based on breast milk, with no addition of water, teas, or any other liquid or semisolid or solid food. Total breastfeeding was defined as the time in months that a child received breast milk (exclusive or not).

Exclusive breastfeeding was divided in 4 categories: ≤7 d, 8 d to <1 mo, 1 to 2.9 mo, and ≥3 mo. Total breastfeeding duration was categorized into 4 groups: <1 mo, 1 to 2.9 mo, 3 to 5.9 mo, 6 to 11.9 mo, and ≥12 mo.

In the 3- and 12-mo follow-ups, mothers were asked whether or not a specific food had been introduced to the child's diet in the past 24 h (in the 3-mo follow-up) or “every day” or “almost every day” (12-mo follow-up), according to a list of food items present in the questionnaire. If so, mothers were then asked the child's age when each food item was introduced for the first time.

All food items introduced in child's diet were classified as liquid, semisolid, or solid, according to their consistency (Supplementary Table 1). Subsequently, the variables age at introduction of semisolid foods and age at introduction of solid foods were categorized into 4 groups: <3 mo, 3 to 3.9 mo, 4 to 5.9 mo, and ≥6 mo.

### Consumption of ultraprocessed foods at 6 y

Between 2010 and 2011, cohort members were followed up at an average age of 6.8 y. Trained fieldworkers collected information about dietary intake using a semiquantitative food frequency questionnaire (FFQ), containing 54 food items divided into 9 food groups.

The frequencies of consumption reported for each FFQ food item were transformed into annual consumption multiplying daily, weekly, monthly, and yearly intake by, respectively, 365.24 d/y, 52.18 wk/y, 12 mo, and 1 y. Food portions were converted into grams to evaluate macronutrients (carbohydrates, protein, and fat) from each food item. Kilocalories were calculated from each food item multiplying carbohydrate and protein by 4 kcal and fat by 9 kcal. Later, yearly energy intake was calculated by summing all the kcals from carbohydrates, proteins, and fats of each food item included in the FFQ. Finally, total daily energy intake was calculated by dividing yearly energy intake of each food item by 365.24. This methodological approach was previously described [22].

The FFQ used in the 6-y follow-up of the 2004 Pelotas birth cohort study was based on three 24-h dietary recalls and was validated in a sample of children ages 1 to 6 y from Pelotas. The unpublished validation study showed a Pearson's correlation of ≥0.50 for macronutrients, calcium, iron, sodium, vitamin C, cholesterol, and saturated fat (unpublished results).

To classify the 54 food items included in the FFQ according to their degree of processing, the food items were categorized into 4 groups based on the NOVA classification [6], [7], [8]. Group 1 contained unprocessed or minimally processed foods; group 2, processed culinary ingredients; group 3, processed foods; and group 4, UPFs. Supplementary Table 2 shows the classification of all food items included in the FFQ according to the NOVA classification.

Kilocalories provided by UPF were calculated by the sum of energy intake from each food. Finally, total daily energy intake from UPFs was divided by total daily energy intake from all foods and multiplied by 100 to obtain the proportion of daily energy intake coming from UPFs.

### Perinatal information

Information about household income at birth (in Brazilian *real*), maternal education (y), self-reported skin color (white, brown, or black), maternal body mass index (BMI) 3 mo after birth, gestational age at birth (mo), birthweight, and child's sex (male or female) were collected at the perinatal interview and were analyzed as potential confounders. We used maternal BMI 3 mo after birth instead pregestational BMI due to high missing values in pregestational BMI information. Nevertheless, pregestational BMI and maternal BMI 3 mo after birth presented high correlation (Pearson's correlation = 0.86) and concordance (Lin's concordance coefficient = 0.82). We classified mothers according to BMI as follows: normal (≤24.9 kg/m^2^), overweight (between 25 and ≤ 29.9 kg/m^2^), or obese (≥30 kg/m^2^).

### Statistical analysis

Analysis of variance was used to assess the association of early feeding practices with the covariables. Crude and adjusted linear regression models were used to assess the effects of early feeding practices (exposures) on proportion of daily energy intake from UPF (outcome), with a significance level of 5%.

To include covariables as potential confounders, a backward stepwise regression was performed, keeping in the model only those variables associated with the outcome at *P* < 0.2. All linear regression models were adjusted for household income at birth, maternal education, and maternal BMI 3 mo after birth. Gestational age, low birthweight, child's sex, and maternal self-reported skin color presented *P* > 0.2 in stepwise regression and were not included in the models.

Variance inflation factor was used to assess collinearity between potential confounders included in the model. All analyses were performed using Stata 13.1 (StataCorp., College Station, TX, USA).

## Results

From the 4231 live newborn infants recruited for the study, 3427 (1772 boys [51.9% of the sample]) were followed at 6 y, had available information for FFQ, and were included in the analyses. Children lost to follow-up belonged to families with lower socioeconomic position at birth (*P* = 0.001) and were born to less-educated mothers (*P* = 0.002). Additionally, children lost to follow-up had a higher prevalence of low birthweight (*P* < 0.001). There were no differences in gestational age, children's sex, and maternal skin color when followed children were compared with those lost to follow-up.

The median exclusive breastfeeding duration was 1.5 mo, and one-fourth of the sample was exclusively breastfed for ≤7 d. The median of total breastfeeding duration was 7 mo, and almost 40% of children were totally breastfed for ≥12 mo. The median age at introduction of semisolid and solid foods was similar (5 and 4 mo, respectively), and <10% of children had semisolid or solid food introduced before 3 mo of age (Table 1).

**Table 1:** Number of children included according to categories of exclusive and total breastfeeding, and age at introduction of semisolid and solid foods (Pelotas, Brazil; N = 3427)

Early feeding practices	N (%)
Exclusive breastfeeding	
≤7 d	859 (25.4)
8 d to <1 mo	375 (11.1)
1–2.9 mo	1211 (35.9)
≥3 mo	931 (27.6)
Total breastfeeding	
<1 mo	358 (10.5)
1–2.9 mo	504 (14.8)
3–5.9 mo	636 (18.6)
6–11 mo	602 (17.6)
≥12 mo	1314 (38.5)
Age at introduction of semisolid foods	
<3 mo	193 (5.8)
3–3.9 mo	497 (15)
4–5.9 mo	1379 (41.6)
≥6 mo	1243 (37.5)
Age at introduction of solid foods	
<3 mo	279 (8.4)
3–3.9 mo	639 (19.2)
4–5.9 mo	1506 (45.4)
≥6 mo	897 (27)

Maximum percentage of unknown observations: (n = 115; 3.4%) for age at introduction of semisolid foods

Children from families with higher income at the time of birth had higher median age for exclusive breastfeeding duration compared with less affluent children. Maternal education and gestational age, birthweight, and being female were positively associated with exclusive and total breastfeeding duration. Furthermore, children who were born to normal or overweight mothers were breastfed longer than their counterparts (both total and exclusive breastfeeding). There were no differences in exclusive breastfeeding according to the children's skin color (*P* = 0.574); however, black children were totally breastfed longer than those classified as white or brown (*P* = 0.018; Table 2).

**Table 2:** Early feeding practices according to socioeconomic, demographic, and maternal characteristics

	Exclusive breastfeeding (mo)	Total breastfeeding (mo)	Age at introduction of semisolid foods (mo)	Age at introduction of solid foods (mo)
	Median (IQR)	Median (IQR)	Median (IQR)	Median (IQR)
Household income at birth (Brazilian Reals – thousands)	<0.001	0.053	0.011	0.451
First (lowest) quintile	1 (0.2; 2.3)	6 (2; 21)	4 (3; 6)	4 (3; 6)
Second quintile	1 (0.2; 2.5)	7 (2.2; 24)	4 (3.5; 6)	4 (3; 6)
Third quintile	1.5 (0.2; 3)	7 (3; 23)	5 (4; 6)	4 (3; 6)
Fourth quintile	1.7 (0.4; 3.3)	7.5 (3; 20)	5 (4; 6)	4 (4; 6)
Fifth (highest) quintile	2 (0.5; 4)	7 (3; 14)	5 (4; 6)	4 (4; 5.5)
Maternal education (y)	<0.001	<0.001	<0.001	<0.001
0–4	1 (0.1; 2)	6 (2; 24)	4 (3; 6)	4 (3; 5.5)
5–8	1 (0.2; 2.5)	6 (2; 20)	4.5 (3.5; 6)	4 (3; 6)
≥9	2 (0.5; 4)	8 (3; 19)	5 (4; 6)	4 (4; 6)
Gestational age (wk)	0.001	<0.001	0.797	0.396
31–36	1 (0.2; 2)	4 (2; 16)	5 (4; 6)	4 (3; 6)
37–38	1.5 (0.3; 3)	6.5 (3; 20)	5 (4; 6)	4 (3.2; 6)
≥39	1.5 (0.2; 3)	7 (3; 22)	5 (4; 6)	4 (3; 6)
Maternal BMI (kg/m^2^)	0.020	<0.001	0.010	0.236
Normal	1.5 (0.3; 3)	7 (3; 21)	5 (4; 6)	4 (3; 6)
Overweight	1.5 (0.2; 3)	7 (2.6; 22)	5 (4; 6)	4 (3.5; 6)
Obese	1 (0.2; 2.8)	4.5 (1.7; 16)	5 (4; 6)	4 (3; 6)
Birthweight (g)	0.001	<0.001	0.714	0.465
<2500	1 (0.2; 2)	4 (2; 15)	5 (4; 6)	4 (3; 6)
≥2500	1.5 (0.2; 3)	7 (3; 21)	5 (4; 6)	4 (3; 6)
Sex	0.024	0.038	0.986	0.512
Male	1 (0.2; 3)	6 (2.5; 20)	5 (4; 6)	4 (3; 6)
Female	1.5 (0.3; 3)	7 (3; 21)	5 (4; 6)	4 (3; 6)
Skin color	0.574	0.018	0.716	0.604
White	1.5 (0.2; 3)	.5 (2.5; 18)	5 (4; 6)	4 (3.5; 6)
Brown	1.2 (0.3; 3)	6 (2.4; 24)	5 (3.5; 6)	4 (3; 6)
Black	1.5 (0.3; 2.9)	9 (3; 24)	5 (4; 6)	4 (3; 6)
Total	1.5 (0.2; 3)	7 (2.7; 21)	5 (4; 6)	4 (3; 6)

BMI, body mass index.

Pearson's χ^2^ (*P*-values) is displayed.

Earlier age at introduction of semisolid foods was associated with lower household income at birth (*P* = 0.011), lower maternal education (*P* < 0.001) and maternal BMI (*P* = 0.010), whereas earlier introduction of solid foods was only associated with maternal education (*P* < 0.001; Table 2).

On average, 40.3% of total daily energy intake at 6 y came from UPFs (643.1 of 1594.7 kcal). Children from less affluent families and those born to mothers with a lower level of formal education received a higher proportion of daily energy intake from UPFs. There were no differences in UPF consumption according to maternal skin color and pregestational BMI, gestational age, low birthweight, and children's sex (Table 3).

**Table 3:** Proportion of daily intake of calories from ultraprocessed foods (in kcal) according to socioeconomic, demographic, and maternal characteristics (Pelotas, Brazil; N = 3427)

	% kcal from UPF
	Mean (SD)
Household income at birth (Brazilian Reals – thousands)	<0.001
First (lowest) quintile	40.9 (12.6)
Second quintile	40.4 (12)
Third quintile	41.9 (12)
Fourth quintile	39.8 (11.3)
Fifth (highest) quintile	38.6 (10.4)
Maternal education (y)	<0.001
0–4	40.4 (12.7)
5–8	41.3 (12.1)
≥9	39.5 (11)
Gestational age (wk)	0.128
31–36	40.8 (12.3)
37–38	39.7 (11.8)
≥39	40.5 (11.6)
Maternal BMI (kg/m^2^)	0.432
Normal	40.7 (11.8)
Overweight	40 (11.6)
Obese	40 (11.6)
Birthweight (g)	0.504
<2500	40.8 (12.1)
≥2500	40.3 (11.7)
Sex	0.433
Boys	40.5 (11.5)
Girls	40.2 (11.9)
Skin color	0.259
White	40.2 (11.7)
Brown	41.1 (11.6)
Black	40.7 (11.9)
Total	40.3 (11.7)

BMI, body mass index; UPF, ultraprocessed foods.

Analysis of variance (*P*-values) is displayed.

Crude effects of early feeding practices on UPF consumption at 6 y are shown in Table 3. The proportion of daily energy intake from UPF was >2 percentage points lower in children exclusively breastfeed for ≥3 mo (β = −2.20; 95% confidence interval [CI], −3.32 to −1.09) when compared with those exclusively breastfed for ≤7 d. Moreover, children who had semisolid and solid foods introduced when they were ≥4 mo also presented lower intake of kcal from UPFs (Table 4).

**Table 4:** Crude coefficients from the linear regression between early feeding practices and ultraprocessed food consumption at 6 y

	Crude model	*P*-value
	β (95% CI)	
Exclusive breastfeeding		<0.001
≤7 d	0.00	
8 d to <1 mo	−0.12 (−1.57 to 1.34)	
1–2.9 mo	−1.04 (−2.09 to 0.02)	
≥3 mo	−2.20 (−3.32 to −1.09)	
Total breastfeeding		0.003
<1 mo	0.00	
1–2.9 mo	0.37 (−1.27 to 2.00)	
3–5.9 mo	−0.22 (−1.78 to 1.34)	
6–11 mo	−1.30 (−2.87 to 0.27)	
≥12 mo	−1.29 (−2.69 to 0.12)	
Age at introduction of semisolid foods		0.004
<3 mo	0.00	
3–3.9 mo	−0.29 (−2.26 to 1.68)	
4–5.9 mo	−2.32 (−4.10 to −0.54)	
≥6 mo	−1.98 (−3.78 to −0.19)	
Age at introduction of solid foods		<0.001
<3 mo	0.00	
3–3.9 mo	−1.18 (−2.85 to 0.50)	
4–5.9 mo	−2.63 (−4.15 to −1.11)	
≥6 mo	−2.68 (−4.28 to −1.09)	

**Fig. 1:**
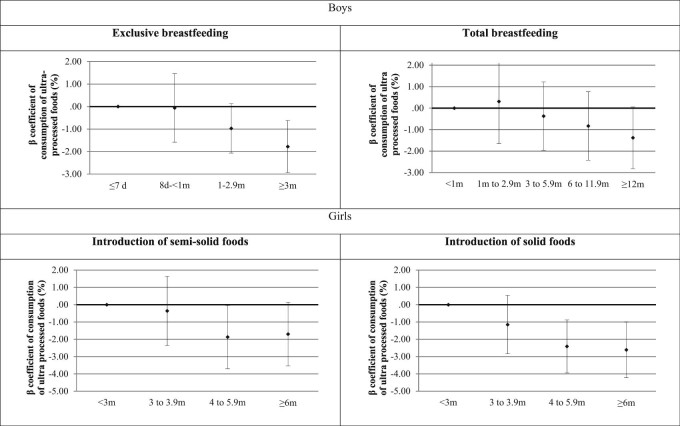
Adjusted association between early feeding practices and ultraprocessed foods consumption at 6 y. Adjusted for household income, maternal education, and maternal body mass index 3 mo after birth.

After adjustment for confounders, exclusive breastfeeding duration and age at introduction of solid foods remained associated with consumption of UPFs. Children exclusively breastfed for ≥3 mo consumed a mean proportion of daily energy intake from UPFs 1.6 percentage points lower than children exclusively breastfed for ≤7 d (β = −1.65; 95% CI, −2.84 to −0.46). Additionally, introduction of solid foods when children were ≥4 mo was associated with a proportion of daily energy intake from UPFs 2.6 percentage points lower when compared with earlier introduction of solid foods (<3 mo; Fig. 1).

## Discussion

Around 40% of total daily energy intake at 6 y of age was provided by UPFs (643.1 of 1594.7 kcal). A recent investigation using data from a representative Brazilian sample aged ≥10 y showed that 21.5% of daily energy intake came from UPFs [23]. These results indicate that, compared with the Brazilian population as a whole, children ages 6 y from Pelotas are consuming a higher proportion of energy from UPFs on a daily basis.

The present study adds evidences of an association of early feeding practices and UPF consumption at 6 y. Exclusive breastfeeding duration and age at introduction of solid foods were negatively associated with UPF consumption, independent of socioeconomic, demographic, and maternal characteristics. However, although association remained significant after adjustment for confounders, the observed effect size was small (<3 percentage points for both exclusive breastfeeding ≥3 mo and introduction of solid foods ≥4 mo). Additionally, it is important to highlight that even with differences between the distinct categories of early feeding practices, the consumption of UPFs was high among all children, with an average of 40% of daily energy intake. Thus, such differences in the consumption of UPFs are not necessarily enough to affect childhood obesity in this population.

Other studies have shown that higher exclusive breastfeeding duration was associated with healthier feeding habits later in life. A recently published study using data from the 2004 Pelotas cohort found that children who were exclusively breastfed for ≥3 mo presented higher adherence to a dietary pattern labeled *fruits and vegetables* at 6 y [24]. Additionally, a study carried out in Canada showed that children who were exclusively breastfed for ≥3 mo presented higher odds of consumption of ≥2 portions of vegetables per day [25].

Despite little evidence regarding the association between age at introduction of complementary feeding and UPF consumption, several investigators have shown that early introduction of complementary foods is related to increased risk for childhood obesity [26], [27]. Additionally, weaned children with high adherence to dietary guidelines in infancy have been found to have higher lean mass in childhood [28]. Other studies have found no association between early introduction of solid foods and later obesity [29], [30], [31]. Differences in the way obesity is measured may be a possible reason for the differences in findings between studies. For example, the use of a *yes or no* variable for obesity in 2 studies [29], [31] may have resulted in loss of power to find significance. Furthermore, differences in sample size, sample age, questionnaires used to record early feeding practices, and analyses models also can be responsible for the heterogeneity of evidence.

Timing of introduction of complementary feeding appears to be related to cardiovascular outcomes in children and adults. A population-based prospective cohort study showed that age at introduction of solid foods was negatively associated with systolic and diastolic blood pressure at 6 y [32]. Moreover, another prospective cohort study observed a positive association between UPF consumption and arterial hypertension in adults [33].

A recent study with individuals from ALSPAC (British Avon Longitudinal Study of Parents and Children) found a lower healthy plate variety score in preschool children who were never breastfed or were breastfed for a short duration, whereas no consistent association between age at introduction of complementary feeding and the score was observed [13].

Two different hypotheses may explain results seen in the present study. The first one is related to the food environment and the influence of parents on children's eating behavior. Eating behaviors depend on the foods offered during the first 2 y of life, an important period in children's dietary transition [34]. Parents or guardians who have higher adherence to healthy guidelines in infancy feeding are more likely to promote healthy eating practices as their children get older, when children's feeding habits are still dependent on parents/guardians' choices, as is the case of this sample of 6 y olds [35], [36]. Thus, breastfeeding duration and timing of introduction of complementary feeding according to guidelines may indicate a greater commitment on the part of parents to support healthy practices as children get older, including reduced consumption of UPFs.

Another hypothesis is that maternal food choices during pregnancy may contribute to children's food acceptance [36], [37]. In this case, early taste might predispose children preferences for salty and sugary foods [14].

The strengths of this study are its population-based cohort design and its large sample size. The use of a standardized food classification [6], [7], [8] also is a strength because it allows comparison with further studies considering the level of food processing. Regular and careful data collection, standardized questionnaires, data quality control, and high retention rates in all follow-ups helped minimize biases.

The unpublished results of the FFQ validation study can be considered a limitation of the present study. Despite the moderate correlation observed between the FFQ when compared with three 24-h dietary recalls, we are not able to reference this validation study because it is not published.

## Conclusions

The present study showed low but positive relationships of exclusive breastfeeding duration and later introduction of solid foods on consumption of UPFs in 6-y-old children, independent of socioeconomic, demographic, and maternal characteristics. Several studies have described the importance of the first 1000 d of life on later outcomes [38], [39], [40]. The present findings are not enough to support causality, but this study reinforces the need for investments in early nutrition and in support of breastfeeding.
