# Supplementary material for: Early feeding practices and consumption of ultraprocessed foods at 6 y of age: Findings from the 2004 Pelotas (Brazil) Birth Cohort Study
Source: Nutrition. 2018 Mar;47:27–32. doi: 10.1016/j.nut.2017.09.012 (PMC5825382; doi:10.1016/j.nut.2017.09.012)
Supplement: Supplementary Table 1 [file mmc1.docx]

**Supplementary table 1.** Classification of foods introduced between 3 months and 1 year of age according to their consistency.

|  | **List of foods** |
| --- | --- |
| Liquids | Whole milk, powder milk, tea, juice, yogurt, bean broth |
| Semi-solids | Paps, porridge and soup |
| Solid | Fruits, egg, bread, cream-cracker or cookie, meat, black bean, rice, pasta and vegetables |
