# Supplementary material for: Early feeding practices and consumption of ultraprocessed foods at 6 y of age: Findings from the 2004 Pelotas (Brazil) Birth Cohort Study
Source: Nutrition. 2018 Mar;47:27–32. doi: 10.1016/j.nut.2017.09.012 (PMC5825382; doi:10.1016/j.nut.2017.09.012)
Supplement: Supplementary Table 2 [file mmc2.docx]

**Supplementary table 2.** Food Frequency Questionnaire food items and their classification according to the processing degree.

| **Processing degree** | **Food items** |
| --- | --- |
| Unprocessed or minimally processed foods | Rice, pasta, potato, cassava, black bean, lettuce, tomato, pumpkin, cabbage, broccoli, raw and cooked carrot, raw and cooked beet, chayote, orange, banana, apple, papaya, tangerine, pear, natural juice, whole and skimmed milk, meat, liver, chicken, fish, eggs and coffee |
| Processed culinary ingredients | Butter, margarine (spread) and icing sugar |
| Processed foods | Bread, cheese and jelly or fruit jam |
| Ultra-processed foods | Cream-cracker, cookies, cakes, yogurt, ham, mortadella, sausage, mayonnaise, candies, chocolate, ice cream, chocolate powder, normal or diet soft drink, artificial juice, chips, sandwich cookies and gelatin |
